# Immune-Modulating Perspectives for Low Frequency Electromagnetic Fields in Innate Immunity

**DOI:** 10.3389/fpubh.2018.00085

**Published:** 2018-03-26

**Authors:** Maria Manuela Rosado, Myrtill Simkó, Mats-Olof Mattsson, Claudio Pioli

**Affiliations:** ^1^Research Consultant in Immunology, Rome, Italy; ^2^SciProof International AB, Östersund, Sweden; ^3^AIT Austrian Institute of Technology, Center for Energy, Environmental Resources and Technologies, Tulln, Austria; ^4^Laboratory of Biomedical Technologies, Division of Health Protection Technologies, ENEA, Rome, Italy

**Keywords:** electromagnetic fields, immune-regulation, damage-associated molecular patterns, inflammation, extremely low frequencies, pulsed electro-magnetic fields, immune system, wound healing

## Abstract

In recent years, the effects of electromagnetic fields (EMFs) on the immune system have received a considerable interest, not only to investigate possible negative health impact but also to explore the possibility to favorably modulate immune responses. To generate beneficial responses, the immune system should eradicate pathogens while “respecting” the organism and tolerating irrelevant antigens. According to the current view, damage-associated molecules released by infected or injured cells, or secreted by innate immune cells generate danger signals activating an immune response. These signals are also relevant to the subsequent activation of homeostatic mechanisms that control the immune response in pro- or anti-inflammatory reactions, a feature that allows modulation by therapeutic treatments. In the present review, we describe and discuss the effects of extremely low frequency (ELF)-EMF and pulsed EMF on cell signals and factors relevant to the activation of danger signals and innate immunity cells. By discussing the EMF modulating effects on cell functions, we envisage the use of EMF as a therapeutic agent to regulate immune responses associated with wound healing.

## Introduction

The immune system is constituted by a very complex network of cells, tissues, and organs that through soluble factors and direct cell-to-cell contacts interact among themselves and with cells belonging to other (organ) systems. This network is the main organizational feature that allows the immune system to keep its dynamic equilibrium (homeostasis) through activating and inhibitory signals and, at the same time, to adapt the response to environmental cues. A healthy immune system permits the organism to interact with the environment in a safe way, keeping invading pathogens under control. At the same time, it “ignores” microorganisms and/or antigens that do not represent a danger for the host.

Perturbing agents, such as toxic compounds (1), ionizing radiation (2), and some pathogens (3) can compromise the integrity of the immune system as they damage immune cells and/or irreversibly alter some immune functions. If the organism is exposed to these factors during early life, when the maturing immune system is particularly susceptible, damages may be immediate, but can also emerge only late in life (4).

Biological effects of the exposure to electromagnetic fields (EMFs) were investigated in a large number of biological targets, including the immune system. The effects depend on frequency, amplitude, and duration of exposure as well as on the characteristics of the targeted cell types. Concerns on possible detrimental effects of extremely low frequency (ELF)-EMF exposure on human health were raised but, due to contradictory conclusions, no consensus was reached (5). Noteworthy, in recent years the possibility to use EMF exposure to modulate immune cell responses has been proposed and debated (6–9). In the present review, focusing on responses to ELF-EMFs and pulsed EMFs (PEMFs), we discuss experimental evidence and unmet issues of this hypothesis, in the context of the current view of the immune system. Nowadays, the immune system is thought to be activated by “danger signals” which are relevant not only to the induction of inflammation and immune responses but also to the activation of counter regulatory (anti-inflammatory/modulatory) mechanisms required to shutdown inflammation and allow tissue healing. While description and discussion of the quoted articles is done in a narrative way throughout the manuscript, the details on exposure conditions of each cited study are summarized in Table 1.

**Table 1:** Available study details in papers cited in sections on danger signals, innate immunity and wound healing, that are dealing with experimental effects of MF exposure.

Reference	Exposure conditions	Model systems	Outcome of exposure

Danger signals			
De Mattei et al. (10)	PEMF; pulse length 1.3 ms; 75 Hz replication rate; 24 h duration; 1.5 mT peak-to-peak induced E-field of 0.07 mV/cm	Bovine synovial fluid fibroblasts	Inhibition of PGE2 production and of enhanced PGE2 release caused by adenosine agonists. Reduced COX-2 expression

Frahm et al. (11)	Sinusoidal MF; 50 Hz; 1.0 mT rms, induced E-field 0.64 mV/cm; exposure duration 5 min–24 h in several steps	Mouse bone marrow-derived macrophages	Increased ROS levels and levels of gp91phox, HSP70, and HSP110 at some, but not all exposure time points

Gottwald et al. (12)	Vertical sinusoidal MF; 50 Hz; 2 µT–4 mT; 15 and 30 min	Human promyelocytic leukemia HL-60 cells, rat heart myoblast H9c2 cells, human Girardi heart muscle cells	Increased expression of HSP72 mRNA during some, but not all exposure conditions. No effects on HSP72 protein levels

Mannerling et al. (13)	Vertical or horizontal sinusoidal MF; 50 Hz; 0.025–0.10 mT rms; 1 h	Human chronic myelogenic leukemia K562 cells	Transient increases in HSP70 protein levels, caused by increased ROS levels

Morehouse and Owen (14)	Vertical or horizontal sinusoidal MF; 6.3 or 8.0 µT rms; 20 min	Human promyelocytic leukemia HL-60 cells	No effects on HSP70 mRNA expression

Pooam et al. (15)	Horizontal sinusoidal MF; 50 Hz; 0.10 or 0.50 mT; 1, 17, or 24 h	Murine RAW 264.7 macrophage cell line	Increased expression levels of the superoxide ion and HSP70 after 24 h exposure

Ongaro et al. (16)	PEMF; pulse length 1.3 ms; 75 Hz replication rate; 1.5 mT peak-to-peak; induced E-field 0.051 mV/cm; 24 h	Bovine synovial fluid fibroblasts	Increased levels of adenosine A2A and A3 receptors. Inhibited release of PGE2, IL-6, and IL-8. Increased release of IL-10

Selmaoui et al. (17)	Sinusoidal MF; 50 Hz; 10 µT; either continuous or intermittent exposure (1 h on—1 h off, where on-cycles contained 15 s on and 15 s off) over-night	32 adult men (20–30 years)	No significant differences on the circadian rhythm investigated on clinical chemistry variables, including uric acid between exposed and sham-exposed groups

St-Pierre et al. (18)	Pulsed frequency-modulated MF or a sequences of short-pulsed (200 ms) “patterned” MF; four intensity levels 5 nT–1.2 µT; prenatal exposure	Albino Wistar rats investigated as 90 days old adults	Elevated uric acid levels in rats exposed to patterned fields

Varani et al. (19)	PEMF; 75 Hz replication rate¸ pulse length 1.3 ms; 0.2–3.5 mT peak-to-peak; peak induced E-field 0.04 mV/cm; 24 h	Human neutrophils isolated from healthy donor’s blood	Increased density and agonist-binding kinetics of membrane-bound adenosine A2A receptors

**Innate immunity—NK cells**			

Bonhomme-Faivre et al. (20)	Sinewave MF; 50 Hz; 0.2–6.6 µT; >8 h/day for 1–5 years	Occupationally exposed workers (*n* = 13)	Increase in NK-cell number

Bonhomme-Faivre et al. (21)	Workers: 50 Hz MF; 0.2–6.6 µT; ≥8 h/day for 1–5 years, followed by 6 months in control environment. Mice: 50 Hz MF; 5 µT; 109 days	Occupationally exposed workers (*n* = 6). Swiss male mice	Workers had increased NK-cell levels compared to control subjects during exposure, non-significant decrease in NK-cell number post-exposure. Mice exhibited decreased NK-cell numbers

Boscolo et al. (22)	Sinusoidal MF; 50 Hz; 0.2–3.6 µT; 40–120 V/m; 20 h/week	Occupationally exposed workers (*n* = 15)	Decreased NK-cell numbers

Del Signore et al. (23)	Sinusoidal MF; 50 Hz; 0.2–3.6 µT; 40–120 V/m; 20 h/week	Female workers occupationally exposed to ELF MF (*n* = 9), both atopic and non-atopic	Decreased NK-cell numbers

Di Giampaolo et al. (24)	Sinusoidal MF; 50 Hz; 0.2–3.6 µT; 40–120 V/m; 20 h/week	Occupationally exposed workers (*n* = 8 female workers; *n* = 7 male workers)	Decreased NK-cell numbers in female workers, no effects in male workers

Gobba et al. (25)	Sinusoidal MF; 50 Hz; low exposure <0.2 μT, high exposure >0.2 μT	Occupationally exposed workers (*n* = 52)	No difference between low and high exposures regarding NK-cell numbers. In workers exposed to >1.0 μT NK-cell lytic activity was decreased

House and McCormick (26)	Sinusoidal MF; 60 Hz; 2 µT, 200 µT, or 1 mT; continuously 18.5 h/day for 13 weeks, 1 mT intermittent 1 h on/1 h off 18.5 h/day for 13 weeks	Female B6C3F1 mice	Decreased NK-cell activity after continuous exposure to 1 mT. No effects of other exposures

House et al. (27)	Sinusoidal MF; 60 Hz; 2, 20, or 100 µT continuously 18.5 h/day for 28 or 90 days, 100 µT intermittent 1 h on/1 h off 18.5 h/day for 28 or 90 days	Male and female B6C3F1 and Balb/c mice, female F344 rats	No effects on NK cells

Ichinose et al. (28)	Sinusoidal MF; 60 Hz; measured during 8 h working shift in three consecutive days	Electric utility worker (*n* = 60)	MF exposure correlated to decreased NK-cell count, no effect on NK-cell activity

Tuschl et al. (29)	Static and LF MF; 500 µT–3 T in MRI environment, 0.01–2 µT by induction heaters; 8 h working day	Occupational exposure in MRI units and at industrial induction heaters	NK-cell count increased among workers at induction heaters

**Innate immunity—neutrophils**			

Bouwens et al. (30)	Sinusoidal MF; 50 Hz or multifrequency “Immunent” signal; 5 and 500 µT; 30 min	Human mononcytic leukemia cell line THP-1	No effects of exposure

Golbach et al. (31)	Sinusoidal MF; 50 Hz or multifrequency “Immunent” signal, 5 and 500 µT; 30 min	Human neutrophil HL-60 or PLB-985 cell lines	No effects on Ca^2+^-signaling in neutrophils

Golbach et al. (32)	Sinusoidal MF; multifrequency “Immunent” signal; 300 µT; 1, 2, 3, or 4 h	Neutrophils isolated from healthy donor blood	Increased extracellular NET-formation in phorbol 12-myristate 13-acetate-stimulated cells

**Innate immunity—macrophages**			

Falone et al. (33)	Sinusoidal MF; 50 Hz; 1.0 mT; up to 96 h	Human SH-SY5Y neuroblastoma cells	Increased levels of antioxidant systems

Frahm et al. (34)	Sinusoidal MF; 50 Hz; 1.0 mT; 24 h	Primary cultures of mouse bone marrow-derived macrophages	Increased IL-1β levels

Frahm et al. (11)	Sinusoidal MF; 50 Hz; 1.0 mT; 5–45 min, 1–24 h	Primary cultures of mouse bone marrow-derived macrophages	Increased ROS levels, transiently increased levels of proteins involved in regulation of redox homeostasis

Gomez-Ochoa et al. (35)	PEMF; 50 Hz burst frequency; 2.25 mT	Fibroblast-like cells isolated from human peripheral blood	Decreased levels of IL-1 and TNF, increased IL-10 levels

Kaszuba-Zwoinska et al. (36)	PEMF; 50 Hz; 45 mT; 3 × 3 h exposures with 24 intervals	Human peripheral blood mononuclear cells from healthy donors and from Crohn’s disease (CD) patients	No effects on cells from healthy donors; cells from CD patients exhibited decreased interferon-γ and increased IL-10 levels

Lupke et al. (37)	Sinusoidal MF; 50 Hz; 1.0 mT; 45 min	Human umbilical cord blood-derived monocytes and human Mono Mac 6 macrophages	Increased ROS levels

Ross and Harrison (38)	PEMF; several frequencies from 5–30 Hz; 4 mT; unknown exposure duration	Mouse RAW 264.7 macrophages	LPS-treated cells exposed to 5.1 and 7 Hz displayed lowered TNF-α levels

Salehi et al. (39)	Sinusoidal MF; 50 Hz; 100 µT; 2 h/day 3 months	Male Wistar ratsIsolated PBMC and spleenocytes from experimental animals	No effects on serum levels of IL-4, IL-6, or IFN-γ. Decreased IL-12 levels.*Ex vivo* PHA stimulated cells from exposed animals had increased IL-6 levels

Selmaoui et al. (40)	Sinusoidal MF; 50 Hz; 10 µT; either continuous or intermittent exposure (1 h on—1 h off, where on-cycles contained 15 s on and 15 s off) over-night	32 adult men (20–30 years)	Increased levels of IL-6 during intermittent, but not continuous exposure. No effects on IL-1β, IL-2, IL-1RA, IL-2R due to any of the exposures

Vincenzi et al. (41)	PEMF; 75 Hz; pulse duration 1.3 ms; yielding a 0.1 duty cycle; peak intensity 1.5 mT; exposure duration unclear	Human neuroblastoma-derived SH-SY5Y cells. Rat PC12 pheochromocytoma cells, N9 microglial cells	Decrease in hypoxia-induced ROS production in PC12, SH-SY5Y, and N9 cells after 24 or 48 h of incubationIn LPS-stimulated N9 cells, PEMF reduced pro-inflammatory cytokines (TNF-α, IL-1β, IL-6, and IL-8)

**Wound healing**			

Callaghan et al. (42)	PEMF; 15 Hz; 4 ms pulse length; max 1.2 mT during pulse; exposure up to 14 days	Db/db (diabetic) and C575L6 (normal) mice with induced dorsal skin wounds	Faster wound healing in both strains due to increased angiogenesis and increased fibroblast growth factor 2 release

Cheing et al. (43)	Sinewave PEMF; 25 Hz; 0.04 ms pulse; max 5 mT during pulse; exposure 1 h daily	Sprague–Dawley rats with streptozotocin-induced diabetes	Accelerated wound closure and re-epithelialization

Choi et al. (44)	Sinewave PEMF; 25 Hz; 0.04 ms pulse; max 5 mT during pulse; exposure 1 h daily	Sprague–Dawley rats with streptozotocin-induced diabetes	Increased collagen fiber deposition in early stages of diabetic wound healing

Delle Monache et al. (45)	Sinusoidal MF; 50 Hz; 1 mT; up to 12 h duration	Human umbilical vein endothelial cells (HUVEC)	Increased endothelial cell proliferation, reorganization of actin fibers, increased expression levels, and phosphorylation of VEGF-receptor 2

Goudarzi et al. (46)	PEMF; 20 Hz, 4 ms, 8 mT; for 1 h per day for 10 days	Wistar rats with streptozotocin-induced diabetes	PEMF increased the rate of wound healing, in diabetic rats

Guerriero et al. (47)	PEMF; unknown frequency with 10.5 GHz carrier wave; 50–100 nW/cm^2^; 20–25 min daily treatment	Case report; two elderly patients with chronic dermal ulcers	Healed ulcers

Ieran et al. (48)	PEMF; triangular pulses; 75 Hz; 1.3 ms pulse length; max flux density 2.8 mT; treatment up to 90 days. Double-blind study	44 patients (28 females, 16 males) with skin ulcers of venous origin	Improved healing

Khooshideh et al. (49)	PEMF; 27.1 MHz; 1,000 pulses/s; 100 µs pulse length; peak power density 75 µW/cm^2^. Double-blind study	Seventy-two female patients undergoing cesarean section	Decreased pain, analgesic use, and surgical wound healing and edema

Lee et al. (50)	Sinusoidal MF; 60 Hz; 0.3 mT. 72 h	CD4+ T-cells isolated from C57/BL6 mice	Upregulation of genes involved in Th17 cell induction, increased differentiation of Treg cells

Loschinger et al. (51)	Sinusoidal MF; 20 Hz; 8 mT; exposure and live-cell analysis during 60 min	Human skin fibroblasts isolated from two individuals	Changes in intracellular Ca^2+^ oscillations

Milgram et al. (52)	PEMF; 5 Hz; 12.5 mT 35–80 J per pulse with 1 µs pulse duration; on days 3, 7, 9, 12, 14, 17, and 22; 1,500 pulses per treatment	Sprague–Dawley male rats	No effects on wound healing

Patruno et al. (53)	Sinusoidal MF; 50 Hz; 1 mT rms; 3 h	Human epidermal keratinocyte HaCaT cells	Increased levels of iNOS, eNOS, NO, AP-1. Increased proliferation. Decreased levels of COX-2, PGE2, catalase, superoxide anion

Reale et al. (54)	Sinusoidal MF; 50 Hz; 1 mT; exposure overnight	Human peripheral blood monocytes	Reduced iNOS expression (mRNA, protein) and activity. Increased MCP-1 expression

Rodemann et al. (55)	Sinusoidal MF; 20 Hz; 6 mT; 2 × 6 h/day, up to 21 days	Human skin fibroblast (HH-8), lung fibroblasts (WI38), SV40-transfromed lung fibroblasts (WI38SV40)	Switch from mitotic to post-mitotic cell populations with increased collagen levels and increased cellular protein levels

Stiller et al. (56)	PEMF; bidirectional 3-part pulse; 2.2 mT; 3.5 ms pulse width; duty cycle 25%. Treatment at home 3 h/day for 8 weeks. Subgroup (*n* = 12) extended treatment with additional 4 weeks. Double-blind study	Patients with full-thickness leg ulcers (*n* = 31)	Decrease in wound surface area, wound depth, and pain intensity. Further improvements in subgroup with extended treatment

Vianale et al. (57)	Sinusoidal MF; 50 Hz; 1 mT rms; 1–96 h exposure duration	Human epidermal keratinocyte HaCaT cell line	Increased growth rate after 48 h exposure. Decreased protein expression levels of RANTES, MCP-1; MIP-1α, IL-8 after 72 h. Decreased NFκB mRNA levels after 1 h

*Studies were performed *in vivo* (animals), *in vitro*, or on patients. PEMF, pulsed electromagnetic field; rms, root mean square; ROS, reactive oxygen species; LF, low frequency; MF, magnetic field; MRI, magnetic resonance imaging*.

## How does the Immune System Perceive Infectious Agents? The Danger/Damage Paradigm

According to the historical self/non-self paradigm, the immune system would generate a response against foreign (non-self) antigens and not against antigens belonging to the organism (self) (58, 59). With time this model was challenged by new findings and revisited. Indeed, the immune system not only recognizes specific antigens (through the antigen-specific receptors of T and B lymphocytes), but also some characteristics or patterns common to groups of infectious agents (pathogen-associated molecular patterns, PAMPs) (60). PAMPs are recognized by germ-line gene-encoded receptors, commonly referred to as pattern recognition receptors (PRR), which are expressed on cells of both the innate and the adaptive immunity. The identification and characterization of toll-like receptors and other groups of PRR, substantiated PAMPs as the initial triggers of an immune response against invading organisms (61, 62). Subsequently, Matzinger (63, 64) proposed that not only external antigens but also self-components, when representing a danger, can trigger an immune response (danger theory). Hence, non-self “safe” antigens would be tolerated by the immune system, as (normally) is the case with commensal bacteria and food antigens.

**Figure 1:**
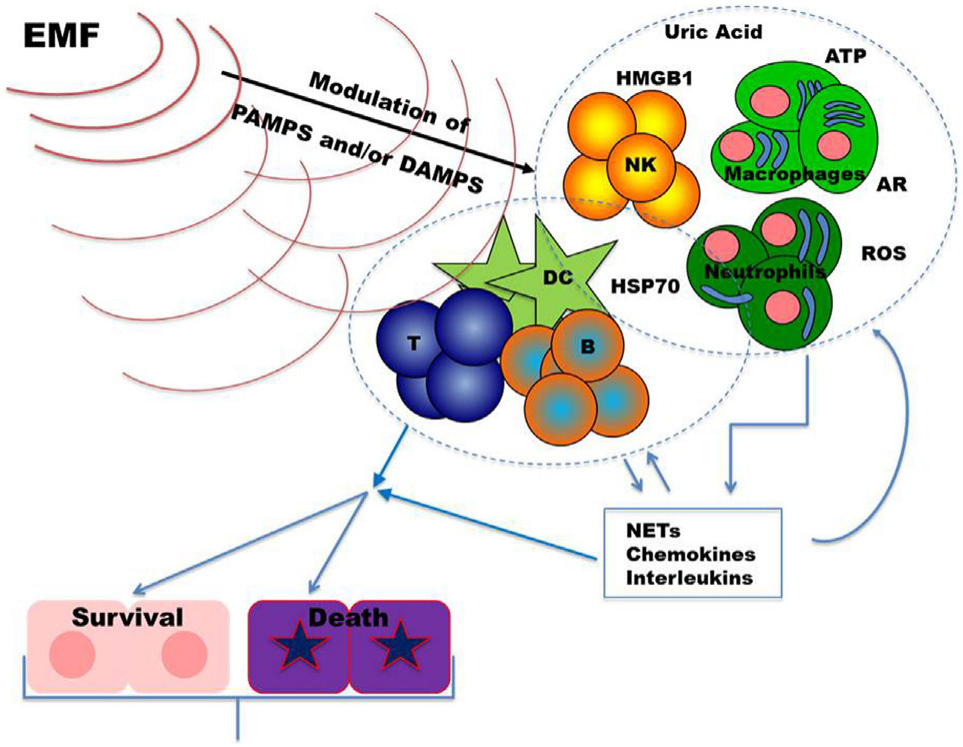
Upon infection and/or tissue damage pathogen-associated molecular patterns (PAMPs) from microorganisms and damage-associated molecular patterns from injured cells alert the immune system. Stimulated innate immune cells, including neutrophils, macrophages, and NK cells, further amplify these danger signals secreting chemokines, cytokines, and other inflammatory mediators. The resulting inflammatory response sustains recruitment and activation of the adaptive immune system cells (T and B lymphocytes). Once an effective immune response is carried out, inflammation returns to homeostatic levels allowing tissue repair. Dysregulations in immune responses lead to chronic inflammation which may result in further tissue damage. Exposure to EMFs could modulate inflammatory responses by targeting, in different cell types, signal transduction pathways and/or molecules relevant to danger signals. Abbreviations: ARs, adenosine receptors; ATP, adenosine triphosphate; B, B cells; DC, dendritic cells; HMGB1, high mobility group box-1; HSP70, heat shock protein 70; NKs, natural killer cells; NETs, neutrophil extracellular traps; ROS, reactive oxygen species; T, T cells.

During tissue damage or cell death a broad array of molecules, defined as damage-associated molecular patterns (DAMPs), is released (Figure 1). DAMPs are very heterogeneous molecules depending on injured cell and tissue types and comprise intracellular proteins (such as heat-shock proteins, HSPs, and high-mobility group box 1, HMGB1), proteins derived from the extracellular matrix (such as hyaluronan breakdown fragments) and non-protein molecules (such as uric acid and ATP). DAMPs can also be actively released by live cells undergoing life threatening stresses in order to signal their status to surrounding (immune) cells. As the PAMPs, DAMPs induce inflammatory processes activating the immune response through the stimulation of PRR (65–67). Thus, these molecules can be used by the organism to alert the immune system for damages induced by invading pathogens, as well as by other noxious agents or “internal” distress. Innate immune cells (neutrophils, macrophages, NK cells, and other cells) exert their first line protective functions and amplify the response by secreting chemokines, cytokines, and other inflammatory mediators. Inflammation promotes cell recruitment, maturation, and activation of the adaptive immune system cells (T and B lymphocytes) which carry out a potent antigen-specific immune response against pathogens (Figure 1).

Alterations in pathways regulating DAMPs generation and their effects can result in inflammatory/immune-mediated diseases. Inhibition of their action may, therefore, represent a therapeutic target in these diseases. Noteworthy, recent evidences revealed that DAMPs contribute to restore the equilibrium (homeostasis) playing an important role also in the promotion of tissue repair (67, 68). As schematized in Figure 1, in the following part of the review, we summarize findings on the effects of the exposure to EMFs on DAMPs, focusing mainly on those relevant to immunomodulation.

## Do EMFs Affect Danger Signals Induction and/or Response?

Heat-shock proteins are highly evolutionary conserved proteins that change their expression levels in response to heat shock, oxidative stress, anticancer drugs, or other stressful conditions [for recent review see Ref. (69)]. HSPs act as molecular chaperones involved in protein folding and transportation. Extracellular HSPs activate antigen-presenting cells (APCs) and take part in the induction of the adaptive immune response by carrying peptides for cross-presentation. HSPs have also been described to act as danger signals (likewise in the absence of pathogens) because, once released in the extracellular milieu during cell damage processes (necrosis), they are able to modulate PAMP-induced responses. As a result, they induce secretion of inflammatory cytokines in APCs, including dendritic cells and macrophages (70).

Not all of the HSPs have an activating, pro-inflammatory function. In some conditions, indeed low amounts of HSPs, such as HSP60, can downregulate adaptive immune responses to self-antigens by upregulating regulatory T (Treg) cell activity and consequently reducing T cell proliferation and counteracting autoimmunity (71). Whether EMFs might be used to induce low levels of HSPs to ameliorate autoimmune diseases has not been investigated. The modulatory role of HSP60 was recently confirmed in wound healing and tissue regeneration. Interestingly, the authors found that HSP60 induced M2 macrophages, a cell type involved in the control of inflammation and regenerative processes (72).

It has been shown that ELF-MF exposure induces HSP expression in mouse macrophages [50 Hz, 1.0 mT up to 24 h (11)] and also in the human leukemia cell line K562 [50 Hz, 0.025–0.10 mT, 1 h (13)]. The authors also detected a possible connection between reactive oxygen species (ROS) release and HSP expression after exposure to MFs (50 Hz, 0.10 mT, for 1 h), since scavengers inhibited the free radical production and the expression of HSP70. It has to be pointed out that none of these studies differentiated between intra and intercellular HSPs. Enhancement of $O_{2}^{-}$ production and HSP70 expression by ELF-MFs (50 Hz, 0.1 or 0.5 mT) were also confirmed in RAW264 cells (an Abelson murine leukemia virus transformed cell line used as a macrophage model) (15). However, in other studies, with exposure of HL-60, H9c2, and Girardi heart cells to lower intensity MF and/or for shorter periods [60 Hz, 6.3 or 8.0 µT, 20 min (14); 50 Hz, 2 µT–4 mT, 15 or 30 min (12)] no change in HSPs expression was detected.

HMGB1 is an archetypical danger signal (alarmin), involved in inflammation-induced tissue damage as well as in tissue repair (73). HMGB1 is released passively during cellular necrosis and actively secreted by inflammatory immune cells (monocytes, macrophages, and dendritic cells) and non-immune cells under stressful conditions (74–76). Oxidative stress-induced HMGB1 secretion is negatively regulated by HSP72 (77–80). Poly(ADP-ribosyl)ation of HMGB1 by PARP1 increases its potential as damage signal. Noteworthy, PARP1 activation is induced by DNA damage and by oxidative stress, and leads to stimulation of inflammation and immune responses (81). In spite of several publications describing the effects of ELF-MFs on oxidative stress and HSPs, no studies reported data on HMGB1 or PARP.

Uric acid is a product of the purine metabolic pathway released by damaged cells and acting as an endogenous danger signal. Uric acid triggers NOD-like receptor protein 3-dependent inflammation, with important implications for systemic inflammatory responses. There are few studies that have measured uric acid in the context of ELF exposure. In human male volunteers, Selmaoui et al. evaluated the effects of acute exposure to both continuous and intermittent EMFs (50 Hz, 10 µT) on the circadian rhythm and on clinical chemistry variables, including uric acid, and found no significant differences between exposed and sham-exposed groups (17). Surprisingly, St-Pierre and colleagues have shown that adult rats prenatally exposed to EMFs [frequency-modulated or sequences of short-pulsed (200 ms) MF, 5 nT–1.2 μT] exhibited high levels of uric acid in addition to various abnormalities of the hippocampus (18). Apart from the fact that the previously discussed publications report different types of EMFs exposures, transient alterations on circulating uric acid levels may reveal damage signals in the form of tissue relevant effects.

Elevated extracellular ATP concentrations represent a damage signal that works as a chemoattractant, induces release of inflammatory cytokines and activates the expression of ectonucleotidases, which rapidly break down ATP to adenosine (82, 83). Adenosine is involved in both upregulation of inflammatory responses and their down-modulation, confirming the dual role of DAMPs-activated pathways in stimulation and control of immune responses. The outcome of the adenosine rise depends on the repertoire of adenosine receptors (ARs) expressed by the targeted cells. Virtually all of the innate and adaptive immune cells, as well as many other cell types, express ARs. Whereas A1 and A3 ARs inhibit adenylate cyclase activity and, therefore, decrease cAMP production, stimulation of A2A and A2B ARs increase cAMP accumulation (84). The cAMP is a potent negative regulator of innate and adaptive immune cells, including effector T cells (85). It induces the expression of CTLA-4, a receptor negatively regulating T cell functions (86, 87), promotes differentiation of regulatory/suppressive T cells and secretion of inhibitory cytokines (88).

There are strong evidences that low-frequency, low-energy PEMFs exert an anti-inflammatory effect through the upregulation of A2A and A3 ARs, leading to a reduction in the expression of inflammatory cytokines (TNFα, IL-1β, IL-8) [(89) and references herein]. PEMF-induced upregulation of A2A receptor occurs in different cell types and tissues, including neuronal cells, osteoblasts, and chondrocytes [reviewed in Ref. (90)]. Neutrophils exposed to PEMFs (pulse length 1.3 ms, 75 Hz replication rate, 24 h, 0.2–3.5 mT) showed significant increase in A2A receptor signaling and in the capability, upon treatment with adenosine agonists, to inhibit the generation of the superoxide anion (19). Of particular interest for the control of ischemic injury are the inhibitory effects of PEMF exposure on hypoxia-inducible factor 1α (HIF1α) expression in microglia cells (41). Indeed, activation of microglia cells during ischemia and reperfusion leads to amplification of danger signals with subsequent strong inflammatory responses that largely contribute to tissue damage. Inhibition of the PGE2 (prostaglandin E2) and cycloxigenase-2 (COX2) pathways, with consequent reduction in the expression of pro-inflammatory cytokines (IL-6, IL-8) and an increase in anti-inflammatory factors (cAMP, IL-10), was also observed in synovial fibroblasts from bovine and osteoarthritis patients (10, 16).

Evidence from the literature thus shows that both ELF-MFs and PEMFs can affect signals associated with damage generally resulting in anti-inflammatory effects. Yet, information on the effects of ELF-MFs and PEMFs on some key damage/danger-associated molecules relevant to immunomodulation, such as HMGB1 and uric acid, is still lacking.

## Effects of EMFs on Innate Immunity

Danger signals may be released by any damaged tissue cell and result in the expression or release of other inflammatory factors by tissue cells and innate immune cells. Innate immune cells represent the first line of defense against pathogens and can actively secrete further danger/activating factors. While producing their response, innate immune cells prepare the context for the activation of the adaptive immune response, which involves antigen-specific receptor-bearing T and B cells. It is, therefore, of interest to consider whether the exposure to EMFs may affect innate immune cells, directly or indirectly through the effects on danger signals or other mechanisms.

### Natural Killer Cells

Natural killer cells (NK) are considered innate immune cells as they express germ-line encoded inhibitory and activating receptors, many in a stochastic way (91). But, they also exhibit characteristics of adaptive immune cells such as the ability to mount enhanced secondary responses to cognate antigenic challenge, revealing their capability to generate memory responses (92). The diversity in pattern expression of NK cells allowed the identification of different subsets performing distinct functions. One of the main tasks of NK cells is to eliminate infected or transformed cells by direct cytolysis or by secreting immune mediators that regulate T cell, neutrophil, and macrophage activation and migration to the injured sites. Thus, altered function of NK cells may strongly impact infection and tumor clearance.

Studies in animal models showed that, in particular experimental settings, exposure to EMFs (60 Hz, 1 mT, continuous, 13 weeks) leads to a reduction or, in more extreme cases to the suppression, of NK activity. Unfortunately, none of these studies demonstrated that short- or long-term exposure to ELF-EMFs would compromise survival upon challenge with an infectious agent or tumor cells in animals whose NK cell function was reduced or lost (26, 27). It is, therefore, difficult to conclude the actual impact on health of these findings. It remains to be addressed if chronic exposure to ELF-EMF would affect cytokine/chemokine production by NK cells and how possible alterations in these soluble factors would interfere with functions of T cells and dendritic cells.

Studies in humans focused mainly on NK cell counts in occupationally exposed subjects (see Table 1) with contrasting results. Some authors observed an increase (20, 21, 29), others a decrease (22–24, 28) of peripheral blood NK cells after ELF-EMF exposure. A study (25) reported the effects of ELF-EMF on NK activity in a group of 52 occupationally exposed workers. Individuals were stratified according to the ELF-EMF exposure dose in low (<0.2 μT) and high (>0.2 μT) exposed workers and NK function assessed as an ability to lyse target cells. Highly exposed workers showed no differences in peripheral blood NK cell number, but a reduction in their lytic capacity compared with low exposed workers. The biological significance of these changes remains to be elucidated.

### Neutrophils

Neutrophils play pivotal roles in recognition, phagocytosis, and eradication of pathogens. Neutrophils, with their wide range of PRR, are able to sense danger and, with their chemotactic receptors, quickly migrate into the sites of injury or infection (93–95). Through the secretion of cytokines and chemokines, neutrophils are able to orchestrate both immune and inflammatory responses. During their action against pathogens, neutrophils may damage tissues, but also support resolution of inflammation and healing of injured tissues (96–98). In addition to the traditional phagocytic function, neutrophils may fight against microbes by delivering granules with enzymatic functions (99) and by what has been defined as neutrophil extracellular traps (NETs) (100). NETs are extracellular traps that capture and destroy extracellular microbes and are constituted by a backbone of nuclear decondensed chromatin mixed with neutrophil-derived antimicrobial proteins. The generation of NET results, for the neutrophil, in a unique form of cell death called NETosis followed by phagocytosis by macrophages (101). NETs are double-edged swords: on one side they limit infection; on the other side they expose self-DNA and intracellular proteins, potentially exposing the organism to the risk of developing autoimmunity and inflammatory diseases (102).

The role that neutrophils play in immune response, their high reactivity, mobility, and sensitivity make them putative targets for investigating possible cell modulation effects by ELF-EMF exposure. The first study investigating ELF-EMF impact on NET formation, *ex vivo*, was performed by the group of Golbach et al. and showed that ELF-EMF (multifrequency “Immunent” signal; 300 µT; 1–4 h) alone was unable to induce NETs (32). However, the ELF-EMF was able to significantly enhance NET formation in freshly isolated peripheral blood neutrophils that were pre-activated with phorbol 12-myristate 13-acetate (PMA). In fact, PMA is a potent activator of NADPH oxidase that triggers the generation of ROS, which are key elements for the induction of NET. ROS is needed for the dissociation of peptide complexes containing neutrophil-derived antimicrobial proteins. Golbach et al. by using selective pharmacological oxidase inhibitors demonstrated that the NADPH pathway is critical for ELF-EMF-enhanced NET formation, possibly by increasing ROS production (32).

Several groups suggested that ELF-EMFs may affect calcium homeostasis and thus interfere in different ways in cell function, depending on the ELF-EMF targeted cell [reviewed in Ref. (103)]. In the case of neutrophils, studies using human promyelocytic cell lines differentiated into neutrophils have shown that an *in vitro* exposure to a 50 Hz sine wave or an “Immunent” signal at an environmentally relevant magnetic flux density of 5 µT or peak 500 µT was not altering the levels of calcium signaling (30). Gene expression patterns of calcium-signaling related genes, cell morphology, as well as the presence of intracellular microvilli were not changed by the MF in either HL-60 or PLB-985 cell lines (31). Thus, under this particular experimental condition, type of ELF-EMF exposure and type of cell lines analyzed, ELF-EMF exposure seems not to interfere with calcium homeostasis in neutrophils.

### Macrophages

Macrophages are cells differentiated from monocytes that left blood circulation and entered tissues, where they work as actuators and regulators of inflammatory processes as well as of innate and adaptive immune responses (104). Macrophages can be activated by the classical (M1 macrophages) or the alternative (M2 macrophages) pathway, resulting in cells producing pro-inflammatory or anti-inflammatory factors, respectively. M1 cells are induced by interferon-γ (IFN-γ), LPS, or endogenous danger signals and produce large amounts of TNF, IL-6, IL-12, and ROS. M2 cells are activated by Th2 cytokines, such as IL-4, IL-10, or IL-13 and act as anti-inflammatory cells. These cells produce low level oxygen intermediates, polyamines and prolines, which induce proliferation and collagen production. The M2 cells are involved in resolving inflammation, increase fibrogenesis, and activate tissue repair and wound healing (105–107). However, while M1- and M2-type factors are relevant elements to define a different activation status of macrophages, the physiological picture might be more complex. Indeed, macrophages do not appear committed to either an entirely inflammatory or an anti-inflammatory expression pattern with some M1 and M2 markers displaying variable expression (108).

Several studies showed that free radical homeostasis is influenced by ELF-EMFs (5, 6, 109, 110). Some *in vivo* studies showed that ELF-MF exposure using different intensities [from 4 mT (PEMF) to 7 mT], time schedules (single or repetitive), and durations (min to days) influence the redox homeostatic system toward a pro-oxidative shift [for reviews see Ref. (110, 111)]. This oxidative stress is very mild, thus no or very moderate cellular/tissue damages, such as lipid peroxidation were detected. However, Lupke and co-workers suggested that ELF-MFs (50 Hz, 1.0 mT, 45 min) would affect immune cells by membrane-associated components leading to moderate ROS release and changes in radical homeostasis. This, in turn, causes down-stream events, including changes in gene expression leading to the activation of the alternative pathway in human monocytes and in a macrophage cell line (37). Under similar MF-exposure conditions (50 Hz, 1.0 mT, different time points), Frahm et al. showed in mouse primary bone marrow-derived macrophages an effect on the expression of redox regulatory proteins associated with increased levels of ROS (11) and IL-1β production (34). Changes in redox status and differentiation were also found in neuroblastoma cells (human SH-SY5Y cell line) after short-term MF-exposure (50 Hz MF; 1.0 mT; up to 96 h) (33). The authors detected modulations of the redox status of the cells, without any oxidative damage, by a positive modulation of antioxidant enzyme expression and a significant increase in GSH (glutathione) levels. It seems that MFs (from 0.025 mT and higher) activate cell systems to release moderate amounts of free radicals *via* the alternative pathway that can lead to the consumption of the intracellular antioxidants. In general, pro-inflammatory factors are downregulated and anti-inflammatory cytokines are upregulated as a result of the moderate oxidative stress induced by MF exposure.

With the aim to investigate the potential for PEMFs to downregulate markers of inflammation expressed by macrophages, Ross and Harrison (38) exposed LPS-activated murine RAW 264.7 macrophages to a square-wave pulsed MF at 0.4 mT. The authors found a statistically significant decrease in the levels of LPS-induced TNF-α and NFκB activation after exposure to 5.1 Hz PEMF, but not at other frequencies (investigated range: 5.1–30 Hz). Unfortunately, this work suffers from partly insufficient description of the exposure conditions and dosimetry and of a large inter-experimental variation for some parameters. Further studies could shed some light on these issues. A reduction in the expression of inflammatory cytokines (IL-1 and TNF) was also observed in PBMC-derived fibroblast-like cells (35) exposed to PEMFs (50 Hz burst frequency; 2.25 mT, 15 min on days 7, 8, and 9 of culture), concomitant with an increase in IL-10 production. In another study (36), the effects of 45 mT PEMF were investigated on cytokine production in PBMC from healthy donors and from Crohn’s disease (CD) patients. Exposed and stimulated PBMCs from CD patients showed a decrease in IFN-γ and an increase in IL-10 production, whereas PEMF-exposure had minimal effect on PBMCs from controls.

Of some interest, for the long *in vivo* exposure period, is the work by Salehi et al. (39), which was performed using male Wistar rats exposed to a 50 Hz sine wave MF (100 µT, 2 h/day, 3 months). There were no effects on body or tissue weight. No effects were found on IFN-γ (a Th1 type cytokine) or IL-4 (a Th2 type) in either serum concentration or supernatants from whole spleen PHA-stimulated cell cultures. A reduction of IL-12 (a Th1-inducing cytokine) concentration in serum, but not in culture supernatants, was reported. Conversely, an increase in IL-6 (an inflammatory cytokine) production by *ex vivo* stimulated spleen cells was observed (but no effects on serum IL-6 concentration). No data were shown on cytokines properly definable as Th17 type. Thus, the authors’ conclusion that ELF-EMFs would downregulate Th1 responses and upregulate Th17 responses is not sustained by the data shown. What cell type was affected by EMF exposure remains undetermined.

The effects of acute exposure to a 50 Hz MF (10 µT) on male volunteers were investigated by Selmaoui et al. (40). Subjects were exposed or sham-exposed at two separate 24 h periods for 9 h during night. The ELF-EMF was either continuous or intermittent (1 h on, 1 h off). Intermittent exposure caused increased levels of IL-6, whereas all the other exposure conditions did not affect IL-6 expression as well as the cytokines and cytokine receptors.

Overall, even if there are some contradictory results especially on the effects on NK cells, several studies show a potential anti-inflammatory effect of the exposure to ELF-EMFs and PEMFs. In macrophages, the reduction in pro-inflammatory cytokines induced by ELF-EMFs is associated with the activation of regulatory mechanisms induced by a moderate oxidative stress. However, in the case of neutrophils, activation of oxidative stress by ELF-EMFs induces activation of NETosis, an effect that might result in a more effective response and, therefore, in the limitation of the inflammatory burden over time. This interesting aspect needs confirmation before any definitive conclusion can be drawn.

## EMF and Immunomodulation in Wound Healing

Wound healing is a very complex process comprising a series of events from initial bleeding and coagulation (hemostasis), to acute inflammation with cell recruitment, proliferation of connective tissue and parenchymal cells, synthesis of extracellular matrix proteins, and finally wound remodeling. The immune cells play a relevant role in all the processes from initial inflammation until tissue remodeling (112, 113). Upon activation, platelets generate the cloth and release several factors which, together with DAMPs from damaged cells and PAMPs from microorganisms, induce an intense inflammatory response. As discussed above, danger signals not only promote inflammation but they also activate processes leading to downregulation of immune responses. This is a very important feature, as control of inflammation is required for tissue regeneration. Alterations in these critical steps result in non-healing ulcers with self-sustaining chronic inflammation as it occurs in some diseases, including neuropathy, ischemia, venous hypertension, diabetes, decubitus, and others (113).

Findings discussed above on the effects of ELFs and PEMFs on inflammation suggest that EMFs could be effective in promoting tissue repair. Indeed, several studies described positive outcomes of EMF exposure in wound healing in both animal models and humans [for review see Ref. (114–116)]. During the initial phases of wound healing, neutrophils and pro-inflammatory M1 type macrophages play a crucial role in the protection from pathogens and in the clearance of cell debris. As already mentioned, exposure of neutrophils to PEMFs promotes the formation of NETs and NETosis *ex vivo* (32), an effect that could potentiate the initial response. Removal of pathogens through an effective action of neutrophils, followed by clearance of dead neutrophils by macrophages, is a prerequisite for the subsequent control of inflammation and repair phases. A switch of M1 to M2 type macrophages (the latter expressing anti-inflammatory and repair-promoting factors) could be promoted by the exposure to ELF-EMF. As discussed above (34, 37), ELF-EMF can modulate ROS in macrophages (and neutrophils) favoring M2 type activation. Low concentrations of ROS may also support healing through promotion of angiogenesis (117). In diabetic mice, exposure to PEMFs (15 Hz; 4 ms pulse length; max 1.2 mT during pulse; exposure up to 14 days) resulted in a faster wound healing associated with an increase in angiogenesis, cell proliferation, and production of fibroblast growth factor 2 (FGF-2). In this model, FGF-2 was also able to prevent tissue necrosis in response to ischemic insult, suggesting that non-invasive angiogenic stimulation by PEMFs might be useful in preventing ulcer formation in diabetic patients (42). Effects on angiogenesis were also found *in vitro* in human endothelial cells exposed to ELF-EMF (sinewave MF; 50 Hz; 1 mT; up to 12 h), with an increase in the expression of VEGF receptor, cell proliferation, and tubule formation (45). Several studies done in rats showed that treatment with PEMFs (20–25 Hz, 0.04/0.4 ms, 4–8 mT, 1 h/day) accelerated wound reduction, enhanced recruitment of myofibroblastic cells, increased collagen fibers and tensile strength, and promoted re-epithelialization in normoglycemic as well as in diabetic animals (43, 44, 46). However, another study did not find effects on skin wound healing in rats exposed to a 5 Hz PEMF with 12.5 mT flux density (52).

Effects of the exposure to ELF-EMF are not confined to innate immune cells. Lee et al. (50) showed that both Th17-type cytokine production and Treg cell differentiation are upregulated in human CD4 cells exposed to 60 Hz 0.3 mT EMF. Noteworthy, Th17 cells play a critical role not only in host defense but also in tissue regeneration, including skin and mucosa (118), whereas Treg cells are deputed to the negative control of immune responses and are required for the maintenance of immune tolerance (119).

Application of PEMFs to the skin was used in various clinical conditions. Reduction in wound healing time and rate of recurrence of chronic venous leg ulcers were described in two randomized double-blinded studies [1.3 ms pulse; max flux density 2.8 mT; up to 90 days (48); 2.2 mT; 3.5 ms pulse; weeks (56)], with beneficial effects and pain reduction lasting beyond the period of treatment. However, in another study, no statistically significant effects were found in healing time of ulcers or pain perception (120). More recently, a reduction of pain and wound inflammation in women undergoing cesarean surgery was reported (49). No adverse effects were observed in any of the studies (47, 48, 56, 120). Although all these *in vivo* studies and clinical observations showed positive results, the real picture is more complex because there is still a fraction of patients that does not respond with improved healing. Several *in vitro* studies, using cell lines or *ex vivo* isolated cells, showed that sensitivity to EMF varies with cell type. In fact, fibroblasts seem to respond better to ELF-EMF of 20 Hz and 6–8 mT (51, 55) whereas keratinocytes (53, 57), monocytes (54), macrophages, and endothelial cells (42, 45) are sensitive to EMF of 50 Hz and 1 mT. Thus, different chronic ulcers, probably depending on their origin, may require different ELF-EMF exposure parameters in order to improve healing.

## Conclusion

Potential use of ELF-EMF and PEMFs as modulator of immune responses alone or in combination with pharmacological therapies represents a novel frontier of investigation with interesting clinical perspectives. As discussed above, danger signals stimulate an immune response, but also activate mechanisms that (later on) will negatively regulate immune cell activation. There seems to be a potential for modulation of danger signals by ELF-MF and PEMFs leading to reduced inflammation (as shown in Figure 1) and promotion of healing processes as indicated by several publications. Mechanisms are not well characterized, but they seem to include increased ROS production and increased expression of certain HSPs for ELF-MFs while PEMFs seem to control inflammation by upregulating ARs pathways. Noteworthy, these pathways are involved in any inflammatory condition and, therefore, they might represent relevant therapeutic targets in several (chronic) inflammatory diseases.

Whereas the majority of the *in vitro* studies focused on monocytes/macrophages and fibroblasts, the effects of the exposure to EMF on other cell types are not well defined. In macrophages, the reduction in pro-inflammatory cytokines induced by ELF-EMF is associated with the activation of regulatory mechanisms induced by a moderate oxidative stress. However, in the case of neutrophils, activation of oxidative stress by ELF-EMF induces activation of NETosis. A better characterization of the effects on neutrophils would be relevant for the treatment of wounds characterized by the presence of infections. Furthermore, studies should also be extended to other key damage/danger-associated molecules to better understand the relations between EMF exposure, oxidative stress, and immune-modulation associated with (wound) healing. Lack of data on Langerhans cells, the skin-specialized dendritic cells, should also be addressed considering their local role in antigen uptake and stimulation of immune responses.

Understanding whether differences in the effects depend on specific exposure parameters or more on targeted cell types, as well as underlying mechanisms, is necessary for possible therapeutic purposes. Addressing this issue needs systematic and comparative studies, where the dependency on waveforms, modulations, frequencies, flux densities, and exposure durations are investigated. Moreover, in order to draw conclusions on possible mechanisms, considering the redundancy of the immune system, studies should always consider the effects on upstream and downstream elements of the investigated pathway rather than just one or two parameters.

Wound healing promoted by EMF in certain patient groups, possibly combined with pharmacological treatments seem to be the most promising area for further studies. However, as discussed above, not all patients experienced a therapeutic effect by the exposure to EMF in wound healing. This observation suggests that for wound healing, EMF could be effective only in some specific types of wounds. ELF-EMFs and PEMFs, by acting through ROS and adenosine pathways, respectively, could thus be suitable for wounds of different origins. Large clinical studies, with well-defined criteria for inclusion of patients, together with experimental studies could shed some light on all these aspects.

## Author Contributions

MR, MS, M-OM, and CP designed the review manuscript structure, drafted, revised, and approved the version to be published as well as agreed to be accountable for all aspects of the work in ensuring that questions related to the accuracy or integrity of any part of the work are appropriately investigated and resolved.

## Conflict of Interest Statement

The authors declare that the review was written in the absence of any commercial or financial relationships that could be construed as a potential conflict of interest.
